# Examining attrition rates at one specialty addiction treatment provider in the United States: a case study using a retrospective chart review

**DOI:** 10.1186/1747-597X-9-41

**Published:** 2014-09-25

**Authors:** David Loveland, Hilary Driscoll

**Affiliations:** Human Service Center, 600 Fayette Street, Peoria, IL 61603 USA

**Keywords:** Engagement, Attrition, Addiction treatment, Healthcare reform

## Abstract

**Background:**

Engaging individuals who have a substance use disorder (SUD) in treatment continues to be a challenge for the specialty addiction treatment field. Research has consistently revealed high rates of missed appointments at each step of the enrollment process: 1. between calling for services and assessment, 2. between assessment and enrollment, and 3. between enrollment and completion of treatment. Extensive research has examined each step of the process; however, there is limited research examining the overall attrition rate across all steps.

**Methods:**

A single case study of a specialty addiction treatment agency was used to examine the attrition rates across the first three steps of the enrollment process. Attrition rates were tracked between August 1, 2011 and July 31, 2012. The cohort included 1822 unique individuals who made an initial request for addiction treatment services. Monthly retrospective reviews of medical records, phone logs, and billing data were used to calculate attrition rates. Attrition rates reported in the literature were collected and compared to the rates found at the target agency.

**Results:**

Median time between request for treatment and assessment was 6 days (mean 7.5) and between assessment and treatment enrollment was 8 days (mean 12.5). An overall attrition rate of 80% was observed, including 45% between call and assessment, 32% between assessment and treatment enrollment (another 17% could not be determined), and 37% left or were removed from treatment before 30 days. Women were less likely to complete 30 days of treatment compared to men. No other demographics were related to attrition rates.

**Discussion:**

One out of every five people who requested treatment completed a minimum of 30 days of a treatment. The attrition rate was high, yet similar to rates noted in the literature. Limitations of the single case study are noted.

**Conclusion:**

Attrition rates in the U.S. are high with approximately 75% to 80% of treatment seekers disengaging at one of the multiple stages of the enrollment and treatment process. Significant changes in the system are needed to improve engagement rates.

## Background

### Attrition is the norm

Engaging individuals who have a substance use disorder (SUD) in specialty addiction treatment services has been and continues to be a daunting challenge for treatment providers. Research has consistently revealed high rates of missed appointments; i.e., no-shows, for assessments and enrollment in addiction treatment services across all levels of care [1, 2] Retaining individuals who eventually enroll in treatment is an additional challenge with attrition rates ranging from 40% to 60% within days to a few weeks of initiating a treatment episode [3–5].

Attrition rates across all stages of the engagement process within an urban specialty addiction treatment provider are reviewed in this manuscript. We first provide a literature review of attrition rates to determine the average rate of withdrawal across each stage of the enrollment process. The review included published reports of attrition rates across each stage of the engagement process as well as the average delay in days between each stage of the enrollment process. These data benchmarks are used to compare the result of our agency data.

### The challenge of engaging people with a substance use disorder

Individuals who seek addiction treatment services represent a small percentage of Americans who have a SUD. SAMHSA [6] estimated that 23 million Americans in 2012 needed some type of intervention to reduce or stop their substance abuse patterns; however, only 16% (3.6 million) of these individuals self-reported a need for treatment. Within the 3.6 million Americans who reported a need for treatment, 69% (2.5 million) received treatment in the past year and the other 31% (1.1 million) did not. Of the 1.1 million who identified a need, but did not receive treatment, 31% (3.41 thousand) attempted to access treatment and the other 69% (7.59 thousand) did not make an attempt.

Individuals who have identified a need for treatment are more likely to be experiencing significant consequences and functional impairments as a result of their drinking or drug use patterns compared to those who have not identified a need for help [7–9]. Individuals who seek addiction treatment are more likely to be male (67% male) [10, 11], unemployed or no longer in the labor force (78%) [12, 13], involved in the criminal justice system (34% to 50%) [12–14] and have had a prior treatment episode (59%) [12] compared to individuals with similar substance abuse patterns who don’t seek addiction treatment services. Individuals who engage in treatment for the first time have been using alcohol or other drugs for 15 to 17 years, on average [15, 16].

### Multiple stages of treatment engagement

Entering a State-funded addiction treatment program involves a three stage process: 1. Initial request for services (IRS) that usually occurs through a phone call, 2. attending and completing the assessment appointment, and 3. being referred to and in enrolling in a treatment program. Completing a treatment episode can be considered a fourth stage. Research has revealed significant attrition at each stage of the process.

Tables 1 and 2 include a summary of published research on the first three stages of the enrollment process. We focused the literature review on evaluations of specialty addiction treatment services reflective of the agency involved in the pilot study described in the method section of this manuscript. Specifically, the review on attrition included:

**Table 1:** **Show rates for clients after IRS or assessment**

Authors & date	# of agencies	Enrollment stage	Sample size	Showed%	No-showed%
Longhi et al., 1991 [17]	Multiple	IRS	1567	71%	29%
Fehr et al., 1992 [1]	1	IRS	505	37%	63%
Festinger et al., 1995 [18]	1	IRS	235	42%	58%
Sequeland et al., 2002* [2]	4	IRS	1777	53%	47%
Chawdhary et al., 2007* [19]	1	IRS	883	42%	58%
**IRS weighted mean**				**54%**	**46%**
**IRS un-weighted mean**				**49%**	**51%**
Longhi et al., 1991 [17]	Multiple	Assessment	909	66%	34%
Kleinman et al., 1992* [20]	1	Assessment	148	58%	42%
Gottheil et al., 1994 [21]	1	Assessment	634	80%	20%
Ershoff et al., 1996 [22]	8	Assessment	1986	80%	20%
Rohrer et al., 1996 [23]	Multiple	Assessment	17,874	45%	55%
Vendetti et al., 1997* [24]	3	Assessment	813	55%	45%
Hser et al., 1998 [25]	Multiple	Assessment	276	62%	38%
Pena et al., 1999* [26]	1	Assessment	294	82%	18%
Weisner et al., 2001 [27]	1	Assessment	1204	76%	24%
Arfken et al., 2001 [28]	1	Assessment	2471	82%	18%
Donovan et al., 2001* [29]	Multiple	Assessment	654	71%	29%
Claus & Kindleberger, 2002 [30]	1	Assessment	260	75%	25%
Parker et al., 2002 [31]	1	Assessment	127	49%	51%
Angarita et al., 2007*! [32]	Multiple	Assessment	372	56%	44%
Pinto et al., 2011* [33]	7	Assessment	286	70%	30%
Resko & Mendoza, 2012* [34]	7	Assessment	340	82%	18%
Molfenter, 2013+ [35]	67	Assessment	?	63%	37%
Pena et al., 1999 (lit review) [26]	22 studies	Assessment	?	73%	27%
**Assessment weighted mean**				**56%**	**44%**
**Assessment un-weighted mean**				**67%**	**33%**

*= randomized clinical trial – secondary analysis; ! = individuals who were accurately matched to treatment were included; + = no show rates were based on enrollment in an outpatient program, but it was unclear if the rate was from IRS or assessment.

**Table 2:** **Retention rates for clients enrolled in treatment**

Authors & date	# of sites	Treatment model	Sample size	Outcome	Retained	Withdrew early
Kleinman et al., 1992 ! [20]	1	Outpatient	86	Retained past 5 sessions	42%	58%
Ershoff et al., 1996 [22]	8	Outpatient	1587	In tx past 30 days	63%	37%
Arfken et al., 2001 [28]	multiple	Residential & outpatient	2026	In tx past 30 days	87%	13%
Claus & Kindleberger, 2002 [30]	1	Residential & outpatient	195	Retained past 2 sessions	80%	20%
Brown et al., 2008 pre NIATx year [36]	1	Residential	279	Retained past 3 sessions	63%	37%
Brown et al., 2008 post NIATx year [36]	1	Residential	674	Retained past 3 sessions	70%	30%
Curran et al., 2007 [4]	> 10	Outpatient	9,933	Retained past 5 sessions	73%	27%
Ghee et al., 2009 [37]	1	Outpatient	104	In tx past 30 days	46%	54%
Adams et al., 2011 [38]	1	Residential	105	In tx past 30 days	77%	23%
Pinto et al., 2011 [33]	6	Outpatient	346	Retained past 5 sessions	83%	17%
McHugh et al., 2013 [39]	1	Methadone Maintenance	78	Retained past 11 sessions	77%	23%
Choi et al., 2013 [40]	3	Residential	1,317	In tx past 30 days	44%	56%
Garnick et al., 2014 [13]	783	Residential & Outpatient	106,662	Retained past 3 sessions	74%	26%
Carroll, 1997 (literature review) [3]	24 studies	Outpatient	>4,000		56%	44%
**30-day retention weighted average without Garnick** [13]					71%	29%
**30-day retention un-weighted average**					68%	32%

- research that focused on engaging clients in a treatment program, such as outpatient, residential, or methadone maintenance (studies that examined transitioning clients from one treatment episode to another, were excluded),
- specialty addiction treatment services, such as non-profit, for-profit, private, public and government-based services (programs housed solely in mental health, medical, university or web-based settings were excluded),
- publications after 1989,
- conducted in the United States only, and
- treatment populations for adults, 18 years and older.

The cutoff of 1990 was selected to include treatment programs that are more likely to reflect specialty treatment programs in 2011 and 2012. Research included naturalistic studies, such as archival data analyses of attrition rates, pre-post interventions designed to improve show rates, and secondary analyses of randomized clinical trials in residential or outpatient settings that examined attrition rates.

Table 1 includes research that examined attrition rates between IRS and assessment, between assessment and enrollment in treatment, or both. The Table includes the manuscript citation, the number of agencies involved in the analyses, the enrollment stage; ie., after IRS or assessment, the number of unique clients tracked in the study, and the percentages of clients who showed and did not show for the assessment appointment or treatment enrollment date. Several studies collected data from centralized intake facilities that referred clients to multiple agencies in a countywide area. The count of “multiple” is used for publications that did not report the actual number of agencies that received clients from a centralized intake program. Weighted and un-weighted mean show/no-show rates are provided for each stage of the engagement process. Publications are listed in ascending order based on publication year within the two categories of IRS and assessment. Pena et al.’s [26] literature review of show rates after assessment is listed at the bottom of the Table, but excluded from the calculation of show/no-show rates. Molfenter [35] did not provide a sample size, so the study was included only in the un-weighted mean estimation.

Table 2 includes published studies that reported early withdrawal rates from either residential or outpatient treatment programs. Two methods were used in the research to measure the 30-day threshold or early withdrawal from treatment for the studies listed in Table 2. Five studies used a specific count of 30 days as the benchmark for early withdrawal. The other eight studies used a specific number of sessions attended as the benchmark for withdrawal before 30 days, with a range of 2 to 5 sessions. The two methods of calculating withdrawal rates are noted under the column “outcome”. Studies are listed in ascending order based on the publication year. Carol’s [3] literature review of retention rates is listed at the bottom of the Table, but excluded from the calculation of show/no-show rates. Garnick et al.’s [13] analysis of 4 state databases of publicly-funded programs and 106,662 clients was also excluded from the calculation of the weighted mean.

Attrition rates across all four stages are summarized in Table 3 based on data from Tables 1, 2 and SAMHSA’s Treatment Episode Data Set (TEDS) for 2010 [40]. Five published studies noted in Tables 1 and 2 that assessed attrition across two or more stages are listed in Table 4. Longhi et al., [17] was the only study we could locate that included an analysis of all four stages (stages 3 & 4 were combined) based on adults calling into a countywide centralized intake program in Seattle, Washington. Arfken et al., [28] tracked clients through stages 2 – 4. Three other studies examined attrition rates across stages 2 and 3, but did not report the number of clients who completed treatment. The percentages displayed in the cells reflect the number of clients from the baseline count that were still in the engagement process after each stage.

**Table 3:** **Summary of attrition rates at each stage of engagement and treatment**

	Weighted mean percentage	Un-weighted mean percentage	Range	Other literature reviews
IRS attrition	46%	51%	29% to 63%	
After assessment attrition	44%	33%	18% to 55%	Pena et al.,1999 [26] –27%
30-day tx attrition	29%	32%	13% to 58%	Carroll, 1997 [3] – 44%
Treatment incomplete SAMHSA [12]	56%	NA	33% to 86%

**Table 4:** **Retention rates across four stages of engagement & treatment**

Authors & date	Sample size	IRS	Assessment	Retained	Treatment complete
Longi et al., 1991 [17]	1567	1118 (71%)	600 (44%)*		386 (28%)
Arfken et al., 2001 [28]	2471		2026 (82%)	1766 (71%)	989 (40%)
Claus & Kindleberger, 2002 [30]	260		195 (75%)	156 (60%)	
Kleinman et al., 1992 [20]	148		86 (58%)	36 (24%)	
Pinto et al., 2011 [33]	346		286 (83%)	201 (58%)	

*= 209 individuals were ineligible for services after assessment, which lowered the base sample to 1358.

Published research on attrition over the past the 24 years has revealed consistent patterns of disengagement as clients were processed through the enrollment steps. Approximately 50% of individuals who made an IRS disengaged before attending an assessment. Approximately 40% of treatment seekers disengaged after completing an assessment and before entering a treatment program. Finally, 30% of all clients who entered an addiction treatment program withdrew or were removed from treatment within 30 days, another 11% to 14% left after 30 days, and an additional 12% to 15% were transferred to a different level of care before completing treatment. Completion rates were higher in residential-based programs and lower for outpatient services. Two studies that tracked a cohort of clients across multiple stages of the engagement process found significant attrition ranging from 60% to 72% between initial engagement and the completion of a single treatment episode.

### Extensive delays between IRS and treatment

Time, as measured in days between each stage of the enrollment process, has been identified as a significant factor of attrition from addiction treatment [5]. Carr and colleagues [41] found that multiple individual, agency and system-level factors contributed to increased wait times between IRS, assessment and treatment enrollment. Attrition as a function of time occurs in two stages: a rapid increase within the first 24 to 48 hours after the IRS, followed by an incremental increase based on each additional day added to the wait time [1, 5, 18].

Individuals who received same day access to an assessment or treatment after making an IRS were significantly more likely to show for their appointment. Five randomized experiments that tested the relationship between wait time and show rates in addiction treatment programs are listed in Table 5. Individuals who made an IRS were randomly assigned to an assessment appointment within 24 hours (same day or by the next day) or to a specific number of days, such as 2, 4 or 7. The average wait in the control condition was 3 to 4 days in four of the five studies.

**Table 5:** **Randomized clinical trials testing same-day access protocols**

Authors & date	Sample size	Same day (0 & 1) show rate	Days 2 – 7	Odds ratio of showing on days 0 or 1
Stark et al. 1990 [42]	38 exp, 22 tau	55% (21)	41% (9)	1.78
Festinger et al. 1996 [43]	39 exp, 39 tau	59% (23)	33% (13)	2.88
Festinger et al., 2002 [44]	58 exp, 58 tau	64% (37)	40% (23)	2.68
Stasiewicz & Stalker, 1999 [45]	32 exp, 96 tau	72% (23)	51% (49)	2.45
Maddox & Desmond 1995* [46]	93 exp, 93 tau	96% (89)	74% (69)	7.74
**Average**		69%	48%	2.56

*Exp* = Subjects assigned to same-day or within 24 hours, *tau* = Treatment as usual_subjects assigned to appointments beyond 24 hours, *= methadone maintenance program-14 days for tau.

Individuals who had same-day access to the assessment after making an IRS were two and half times more likely to show, on average, compared to individuals assigned an appointment that was 3 to 7 days from the IRS. The odds ratio would have been larger if researchers had extended the wait time in the control condition beyond seven days, as was shown in the Maddox and Desmond [46] study. Several pre-post or naturalistic studies found similar results [1, 17, 18, 30, 47].

Table 6 includes 12 studies that reported an average number of days between IRS and assessment, between assessment and enrollment in treatment, or both. Publications included six listed in Tables 1, 2, 3 as well as six additional studies that specifically examined waiting times or wait lists in specialty addiction treatment programs. Articles are organized by IRS or assessment stages and listed in ascending order by publication year.

**Table 6:** **Average days between IRS & assessment and assessment & treatment**

Authors & date	# of agencies	Sample size	Tx type	Stage of enrollment	Average # of days
Longhi et al., 1991 [17]	Multiple	1567	Residential & Outpatient	IRS	21
McCarty et al., 2007* [48]	13	6016	Residential & Outpatient	IRS	5
Carr et al., 2008 [41]	Multiple	577	Outpatient	IRS	4.4
Brucker & Stewart, 2011 [49]	Multiple	6629	Outpatient	IRS	8.1
Brucker & Stewart, 2011 [49]	Multiple	2457	Intensive Outpatient	IRS	7.9
Quanbeck et al., 2013 [50]	192	NA	Residential & Outpatient	IRS	7.2
**Weighted average # of days between IRS & assessment**					**8.0**
**Un-weighted average # of days between IRS & assessment**					**8.9**
Hoffman et al., 2011 [5]	15	4937	Residential & Outpatient	Assessment	8.3
Donovan et al., 2001 [29]	Multiple	654	Residential	Assessment	24.2
			Outpatient	Assessment	9.9
Arfken et al., 2001 [28]	Multiple	2026	Residential & Outpatient	Assessment	9.1
Claus & Kindleberger, 2002 [30]	1	260	Residential & Outpatient	Assessment	4.2
Downey et al., 2003 – women only [47]	Multiple	206	Residential & Outpatient	Assessment	29
Downey et al., 2003 – men only [47]	Multiple	448	Residential & Outpatient	Assessment	16
Chawdhary et al., 2007 [19]	1	883	Outpatient	Assessment	6.2
McCarty et al., 2007* [48]	13	6016	Residential & Outpatient	Assessment	10
Brucker & Stewart, 2011 [49]	Multiple	6629	Outpatient	Assessment	4.9
Brucker & Stewart, 2011 [49]	Multiple	2457	Intensive Outpatient	Assessment	3.9
Guerrero, 2013 [51]	104	13,329	Residential & Outpatient	Assessment	3.4
**Weighted average # of days between assessment & treatment enrollment**					**6.4**
**Un-weighted average # of days between assessment & treatment enrollment**					**10.8**

*NA* = Not applicable, sudy did not track people, but rather tested the wait time after IRS by making calls to 192 agencies; *= baseline data before NIATx changes occurred.

The average wait time between IRS and assessment was 8 to 9 days (range 4 to 21 days) and the average wait time between assessment and treatment enrollment was 6 to 11 days (range 3 to 24). Wait time for residential treatment was usually longer than outpatient services, when both levels of care were assessed within the same study.

There is an extensive body of literature on attrition rates at each step of the enrollment process; however, we located only two publications in the past 25 years that tracked a cohort of treatment seekers over three or four steps or stages of the enrollment process. Cross sectional data and limited longitudinal data indicated extensive attrition of treatment seekers who make an IRS. The purpose of this study was to track a cohort of clients across the first three stages of the enrollment process to determine the true attrition rates when all steps are included.

### Case study of a specialty behavioral health agency

The attrition rates of a large, specialty addiction treatment provider were examined and compared to rates collected from our literature review. Retrospective medical records data were used from the target agency to examine the attrition rate of individuals seeking addiction treatment. Tracking of clients was initiated on August 1, 2011 when the target agency expanded outpatient services and closed one of its three long-term residential programs. Prior to the change on August 1, 2011, the agency provided outpatient services to men and women who were in either criminal justice-based programs or medication-assisted services, but had no general outpatient services for voluntary clients who were not in need of either a residential level of care or medication-assisted treatment. Starting on August 1, 2011, the agency expanded outpatient addiction treatment services by adding approximately 100 additional treatment slots for men and women who were not involved in the criminal justice system while maintaining the other outpatient services. The new outpatient model was based on a cognitive behavioral therapy format [52]. The agency also closed a long-term residential program for women (24 beds), leaving two remaining residential programs, one for men (32 beds) and one for women (28 beds). All residential programs were 60 to 90 days in duration.

Identifying the overall attrition rate was the primary purpose of tracking all individuals who requested treatment at the target agency for 12 consecutive months, beginning on August 1, 2011.

## Methods

### Target agency

The agency, heretofore referred to as Urban Addiction Treatment (UAT), is nested within a city of 120,000, a county of 190,000, and a three-county area of 350,000 residents in Illinois. Racial mix of the city includes 73% White, 20% African American, and 4% to 6% Hispanic populations. Demographic indicators of the city highlight a population that has a significantly high poverty rate, compared to the State average, along with other commonly linked conditions associated with high poverty; e.g., high rates of infant mortality, crime, addiction, HIV/Hepatitis, and other medical conditions.

UAT is classified as a 501c-3, private, nonprofit, specialty behavioral health organization that provides a range of treatment interventions on an annual budget of approximately $16.5 million dollars in the 2011–2012 fiscal year. UAT provided a range of addiction treatment services to 2,000 to 3,000 individuals annually in years 2011 and 2012, which included:

- a 12-bed medical detoxification program,
- gender specific residential programs,
- intensive (IOP) and low-intensive outpatient (OP) services,
- medication assisted treatment (methadone & suboxone),
- treatment services for clients enrolled in a two problem-solving court programs: one for drug court and one for mental health court,
- State-licensed DUI assessment and treatment services,
- ancillary services (e.g., psychiatric consultation, HIV testing, transportation services), and an outpatient program that serves clients from Employment Assistance Programs (EAPs) and private insurance.

UAT is also the County’s community mental health center, serving approximately 1,000 adults with a serious mental illness annually. UAT is a safety-net organization in terms of the target population. Individuals who receive UAT services were and still are highly likely to be unemployed, involved in the criminal justice system, receiving public assistance in the form of Medicaid and social security disability benefits (or indigent), and have received prior specialty services at UAT or other agencies in the State.

UAT’s service profile was unique during the cohort tracking period in several aspects compared to other specialty addiction treatment providers in the State as revealed in SAMHSA’s National Survey on Substance Abuse Treatment Services [40, 53]. Unique or uncommon aspects of UAT’s service profile included:

- free standing medical detoxification unit,
- active daily census of over 200 clients and larger caseloads in detoxification and residential treatment than national averages,
- two long-term residential programs (60 to 90 days),
- a comprehensive range of mental health services for people with serious mental illness,
- Joint Commission Accreditation (most specialty addiction treatment agencies in the U.S. are accredited by CARF),
- Onsite primary care clinic operated by a Federally Qualified Health Clinic, and
- medication assisted treatment for methadone and suboxone.

The catchment area of addiction treatment services includes the entire State of Illinois. Between 40% and 60% of clients enrolled in the residential programs resided outside of the primary county where UAT is located. The outpatient programs tended to serve individuals living within a three county area.

UAT’s funding structure is similar to most non-profit specialty behavioral health programs in the U.S. in that it relies on State general revenues, federal block grant dollars, and Medicaid to finance services [30, 37]. The Division of Alcohol and Substance Abuse (DASA), within the Illinois Department of Human Services (IL-DHS), is the primary customer of UAT’s addiction treatment services and provide approximately 90% of UAT’s revenues for addiction treatment services. DASA provides funding to UAT through an annual capped contract. DASA dollars include a combination of state general revenues and federal block grant dollars. UAT can also bill Medicaid or private insurance, in place of DASA funds, for those clients who have insurance. DASA contract dollars and State Medicaid are earned through a fee-for-service billing process.

UAT developed a centralized intake process to manage the large flow of individuals seeking mental health or addiction treatment services. The benefit of this process was instituting a uniformed assessment that reduced data collection errors while meeting the unique requirements of DASA, the Division of Mental Health (DMH), and the Joint Commission. The downside of the centralized process was an expanded paperwork protocol that added survey questions and time to the assessment. Individuals had to complete an assessment process that required one to two hours of their time before an appointment for treatment could be established.

### Procedures

Individuals were included in the cohort if they had

- an IRS through UAT’s central intake program between August 1, 2011 and July 30, 2012,
- requested addiction treatment services during their IRS,
- agreed to be scheduled for an assessment (phone or in person), and
- were 18 years or older.

The cohort was identified through a review of medical records and an in-house database program that stored information on all IRS calls. A 30-day timeframe was used within each stage of the enrollment process to categorize individuals in the cohort based on their referent request for treatment. IL-DHS/DASA established a 30-day window for adults enrolled in an outpatient program [54]. The outpatient addiction treatment episode was terminated if an individual did not receive a billable contact within 30 days. For consistency, we used the same 30-day period to categorize individuals who requested treatment and were waiting for an assessment as well as for those who completed the assessment and were waiting to enter a treatment program.

### Measurement

Show/no-show rates were tracked through the first three stages of the enrollment process. Measurement of each stage included:

### Initial request for services (IRS) attrition rate

Individuals who received an assessment within 30 days of the referent IRS were classified as attending/showing for their appointment and counted once, even if they had scheduled and missed several appointments between the request and completing the assessment. Individuals who did not attend the assessment appointment within 30 days were classified as missing the assessment once, even though many of these individuals scheduled and missed two or more appointments in 30 days. Between 30% and 50% of all assessment appointments were rescheduled based on individuals missing their appointments (not calling in advance) or calling and rescheduling.

### Assessment attrition

Individuals classified as attending/showing for their assessment were tracked for up to 30 days from the date of their assessment. Individuals were classified as either attending/showing for treatment enrollment or no-showing if they did not enter a treatment program within 30 days. Billing data were used to determine if a person showed for treatment.

### 30-day treatment attrition

Individuals who enrolled in treatment were tracked for 30 days from the treatment enrollment date. Two protocols were used to measure attendance/attrition over 30 days based on level of care. Individuals enrolled in residential treatment, excluding detoxification, were classified as actively engaged in treatment if they received 30 continuous days of treatment and were actively enrolled on the 30^th^ day, based on billing records. All individuals who were discharged prior to the 30^th^ day were classified as early withdrawal. NIATx guidelines were used to measure attrition in outpatient services [48]. Individuals who enrolled in the outpatient program were classified as attending treatment if they received four treatment episodes, delivered on four separate days, within 30 days of the treatment enrollment date. A treatment episode included either individual or group counseling session (nearly all contacts were group sessions). Individuals who received three or fewer billed events within 30 days of being enrolled in the program were classified as early withdrawal, even if they received a billable contact beyond 30 days (e.g., reengaged before being closed).

### Addiction treatment services tracked

All UAT addiction treatment services were included in the tracking process, such as residential treatment, medication-assessed OP, court-based services, or the newly formed OP program. Individuals seeking only detoxification or needing to complete a State-licensed DUI-specific assessment and education program without requiring additional treatment options were excluded from the cohort.

### Demographic data collected

Demographic data were not available for most of the individuals who made an IRS, but did not show for the assessment; so only the count of IRS’ made was included in the first stage of the enrollment process. Demographic data were collected on all individuals who completed the assessment and pulled from UAT’s electronic health record or chart reviews. Age, gender, and race were consistently reported (entered) in the medical record and were used in the analyses. Other demographics, such as income, employment status, or marriage status were inconsistently reported and were excluded from analyses.

Treatment history and involvement in the criminal justice system were pulled from chart reviews of the assessment form. History of addiction treatment was converted into a dichotomous variable of 1 for any prior addiction treatment or 0 for none noted in the assessment form. Criminal justice involvement was grouped into three categories: 1. No involvement noted in the assessment form, 2. on probation or parole, or 3. involved in Department of Child and Family Services (DCFS).

A research associate pulled data monthly from an agency database that stored the names and contact information of all individuals who called UAT requesting treatment. Contact data were reviewed with the intake staff on a monthly basis to verify information. Billing data were pulled from the agency’s electronic health record system that included both service and billing data. The University of Illinois College of Medicine at Peoria’s Institutional Review Board provided approval for the retrospective chart review protocol.

## Results

### Descriptive analyses

The total cohort included 1822 individuals who made an IRS and were given an assessment appointment. Table 7 displays the demographic data of 1003 clients who completed the assessment. The cohort was mostly White (78%), more likely to have had one or more prior treatment episodes (64%), slightly less likely to be involved in the criminal justice system (44%), nearly split between men and women (52/48), and averaged 34 years old. Demographic data are displayed across the assessment, treatment enrollment, and 30-day treatment retention stages. An odds ratio of completing 30 days of treatment was calculated for each demographic variable noted in the Table. Women were less likely to complete 30 days of treatment compared to men (OR = .73; CI 95% .60 to .86). There were no other significant or differential attrition rates found for prior treatment experience, age, race, or involvement in the criminal justice system.

**Figure 1:**
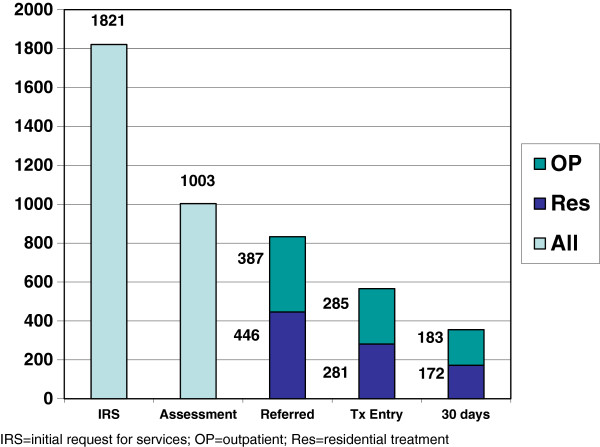
**Attrition rates by level of care and stages of enrollment.**

The overall attrition rates by each enrollment stage are displayed in Figure 1. Forty-five percent of individuals who received an IRS did not show for the assessment appointment. Thirty-two percent of individuals who completed the assessment and were referred to treatment did not enroll in a treatment program and another 17% were not referred or the referral destination could not be determined. Thirty-seven percent of individuals who enrolled in treatment withdrew before completing 30 days of treatment. Overall, 80% of the cohort disengaged from the enrollment process before completing 30 days of treatment.

**Table 7:** **Demographic data across enrollment and treatment stages**

	Assessed for treatment	Enrolled in treatment	Stayed in treatment 30 days
**Gender**	**1003**	**577**	**362**
Male	522 (52%)	310(54%)	206(57%)
Female	481 (48%)	267(46%)	156(43%)*
**Race**			
White	739 (74%)	451 (78%)	283 (78%)
Black	185 (18%)	114 (20%)	74 (20%)
Other	24 (2%)	12 (2%)	5 (1%)
Unknown	55 (5%)	0 (0%)	0 (0%)
**Age**			
Average Age	34	34	35
**Treatment history**			
Prior treatment	587 (59%)	371 (64%)	235 (65%)
No prior treatment	327 (33%)	199 (34%)	123 (34%)
Unknown treatment history	89 (9%)	7 (1%)	4 (1%)
**On probation or parole**			
Criminal justice involvement	386 (38%)	253 (44%)	167 (46%)
No criminal justice involvement	538 (54%)	318 (55%)	191 (53%)
Unknown criminal justice status	78 (8%)	5 (1%)	4 (1%)
**Open case with DCFS**			
Involved with department of children & family services (DCFS)	104 (10%)	54 (9%)	32 (9%)
No CFS involvement	812 (81%)	517 (90%)	326 (90%)
Unknown CFS status	87 (9%)	6 (1%)	4 (1%)

*= Odds Ratio of .83 (CI 95%, .6 to .96).

For clients who showed for the assessment or enrolled for treatment, the median time between IRS and assessment was 6 days (mean 7.5) and the median time between assessment and treatment enrollment was 8 days (mean 12.5). Treatment enrollment appointments were not established for clients referred to residential treatment because all clients were placed on a waiting list; so we were unable to determine the median time between those who showed and did not show for treatment.

Table 8 includes data on the outcomes of clients based on the initial referral to treatment; i.e., OP or residential. Seventeen percent (170) of the clients assessed were either not referred to treatment for a variety of reasons (97; e.g., waiting for court hearing or not wanting additional services), referred out (28) or unknown (45). However, 11 of these individuals eventually arrived at a treatment program as noted in the Table and were included in all analyses. For clients who enrolled in treatment, 97% of those referred to OP arrived at OP (3% switched to residential) and 87% of those referred to residential arrived at residential (13% switched to OP). Sixty-three percent (362) of all clients who enrolled in treatment remained in treatment for 30 days with similar rates of retention between OP (4 or more billable contacts within 30 days) and residential levels of care.

**Table 8:** **Client outcomes based on residential or outpatient referral**

Referral at assessment	Total referred (%)	Did not show for tx or not referred (%)	Showed for residential (%)	Showed for OP (%)
Residential	446 (44%)	131 (31%)	273 (96%)	42 (14%)
OP	387 (39%)	136 (32%)	8 (3%)	243 (83%)
Not referred or unknown	170 (17%)	159 (37%)	2 (1%)	9 (3%)
**Total**	**1003 (100%)**	**426 (100%)**	**283 (100%)**	**294 (100%)**

We completed a prospective review of UAT’s billing records up to June 30, 2014 for the 1003 clients who had completed an assessment during the cohort tracking period and found that:

- 233 (23%) had at least one more IRS during the 12-month tracking period,
- 286 (29%) had an IRS and assessment completed over the following two years,
- 249 (25%) received one or more UAT addiction treatment episodes after the referent treatment episode in the cohort, and
- 145 (14%) enrolled in UAT’s detoxification program one or more times after their referent contact in the cohort.

The final analysis is a comparison of UAT’s attrition rates with the averages noted in Tables 3, 4, 6 and 9. Longi et al.’s [17] results are included in the last column. We calculated an estimated number of clients who would still be in treatment at 30 days based on the average attrition rates listed in each column, with all four columns starting at 1822 clients. UAT’s final count based on actual clients is lower; i.e., higher attrition, than the estimated counts listed in the other three columns due to UAT’s higher attrition rate during treatment. UAT’s attrition estimate may also be higher due to the unknown outcome of 170 individuals who were assessed, but a referral was not made or could not be determined. UAT had a similar mean wait time between IRS and assessment, but a longer mean wait time after assessment and before treatment enrollment compared to the averages derived from the literature review.

**Table 9:** **Comparison of UAT’s attrition rates with published research**

Stage of assessment	UAT	Literature review un-weighted mean	Literature review weighted mean	Longi et al., 1991 [ [17]]
IRS to assessment	45%	51%	46%	29%
Assessment to treatment	32% + 17%	33%	44%	44%
Treatment enrollment to 30 days retention	37%	32%	29%	36%*
Total count remaining at 30-days in treatment	**362**	**407!**	**391!**	**463!**
Mean wait time IRS to Assessment in days	7.5	8.9	8.0	NA
Mean wait time assessment to treatment enrollment in days	12.5	10.8	6.4	NA

*= completed treatment, could be a high estimation for 30-day attrition; ! = extrapolated from percentages.

## Discussion

The use of a single case study provided an opportunity to examine the overall attrition rates for individuals seeking addiction treatment over a period of 12 months. However, UAT’s attrition rates are based on one agency and may not be indicative of approximately 355 nonprofit, 257 for-profit or 26 government-based organizations providing addiction treatment services in Illinois [53]. Another limitation of the study is that attrition rates and demographic data were based on retrospective chart, contact and billing data rather than on prospective tracking of individuals. Medical charts, electronic records and billing records are prone to entry errors and omissions. Establishing a referent appointment date after the IRS was particularly challenging. Individuals frequently cancelled and rescheduled appointments before the assessment occurred. We encountered numerous data entry errors as a result of the rescheduling process; therefore, we abandoned the task of collecting scheduled appointment dates and selected the actual date of the assessment, once it occurred and was billed to the State funder. Classifying individuals who were not referred to treatment was another dilemma. We identified 170 individuals (17% of those who completed the assessment) who did not receive a clear referral to a UAT addiction treatment program; however, 11 of these individuals eventually enrolled in treatment during the 30-day window of tracking. Moreover, the assessment staff frequently noted in writing that these no-referral clients were waiting for a court hearing before deciding if they would proceed to treatment. In other words, many of these individuals were not ready to enroll in treatment, but were keeping their options open in case they were court ordered to treatment. Finally, the attrition rate for clients in outpatient services was slightly inflated due to our benchmark of four billable events within 30 days based on NIATx guidelines. Some of the clients who received three or fewer billable events within the 30-day timeframe received additional services beyond 30 days.

With these limitations noted, UAT’s rates of attrition were similar to those found in the literature after the IRS (UAT 45%, literature 29% to 51%), after the assessment (UAT 32% to 49%, literature 33% to 44%) and somewhat higher for the first 30 days of treatment (UAT 37%, literature 29% to 36%). The waiting time between IRS and assessment was also similar to the rate found in the literature review (UAT 7.5, literature 8.0 to 8.9); whereas the waiting time between assessment and treatment enrollment was slightly higher than the average found in the literature (UAT 12.5, literature 6.4 to 10.8).

UAT’s population mix was similar to the national average of treatment seekers in age (34, national average = 34), percentage of clients with prior treatment experience (64%, national average = 59%), and percentage of clients involved in the criminal justice system (44%, national average = 30% to 50%). UAT’s population had a higher percentage of women (48%, national average = 33%) and a higher percentage of White, non-Hispanic clients (78%, national average = 60%) seeking treatment services [40].

A substantial amount of UAT resources and State dollars were used to engage clients with limited success. Many of the clients cancelled and rescheduled appointments, which extended the waiting time between enrollment steps. UAT engagement staff were flexible and accommodating of clients; however, each rescheduled appointment filled up another assessment slot of one to two hours as well as around 10 minutes of phone time to acquire information (e.g., insurance status, involvement in criminal justice, appointment time, & providing directions to the facility). It was common to see several hours of staff time dedicated to completing one client assessment, if no-shows and phone time were included in the calculation.

Treatment shopping for residential services was a technique used by some clients trying to enter a State-funded program. Clients had to wait two or more weeks after their assessment before knowing when they would enter a UAT residential treatment program. The process of waiting for a residential enrollment date was and still is the common practice for State-funded treatment programs in Illinois. Clients would complete an assessment at two or more agencies, without revealing the information to UAT staff, in the hope of gaining an earlier entry date at one of the facilities. Provider shopping added names to wait lists across multiple agencies and redundant assessments, while decreasing the likelihood that a client would enroll in a UAT residential treatment program.

Further, 37% of clients left treatment incomplete within 30 days of enrolling, with similar rates observed in residential and outpatient services. One out of every four clients in the cohort returned to the UAT engagement process within 12 months of their referent contact, seeking additional treatment services. Each new episode required the completion of all three enrollment steps. Nearly two thirds of the cohort had received one or more prior treatment episodes at UAT or other agencies, but none of the 1822 clients were involved in a continuum of care when they contacted UAT during the cohort tracking period.

Our study confirmed that most individuals drop out of the enrollment process before receiving a sufficient dosage of addiction treatment. The next step is to develop interventions that improve the engagement process through the front door as well as the back door as clients’ transition through various levels of care. Multiple research studies have shown that people provided with same-day access for an assessment are two to three times more likely to show for their appointment compared to people given an appointment within three days of their IRS. It is not surprising to see attrition rates around 50% between IRS and assessment when the average wait is 7 to 9 days in most addiction treatment agencies, including UAT. However, there is limited research on how to effectively triage clients who show for same-day assessments. UAT had the staff resources to provide same-day assessments, but lacked the capacity to provide treatment on demand to a larger pool of clients resulting from the same-day assessment process. Moreover, there is limited research on how to engage people who are not ready for the limited menu of treatment options provided by the specialty addiction treatment system.

## Conclusion

Engaging and effectively treating individuals with a substance use disorder remains a challenge in the specialty addiction treatment field. One out of five individuals who contacted UAT for addiction treatment services received a minimum of 30 days of residential or outpatient services. The overall attrition rate of 80% was high, but within the range found in the literature (75% - 79%). Limited treatment slots at UAT were the primary reason why people had to wait three weeks, on average, before receiving treatment services. Nonetheless, it is unclear if people disengaged because of an increased ability to manage their SUD on their own, low readiness for treatment, the long wait for treatment, dissatisfaction with the treatment options, complications associated with their addiction that occurred during the wait, or an interaction of these factors over time. The Accountable Care Act (ACA) will increase the number of people who have insurance for behavioral health services, which in turn could increase addiction treatment slots [55–57]. Increased funding is essential, but may not be sufficient to resolve all the factors associated with the high attrition from treatment at UAT and most specialty addiction treatment providers in the U.S. Capoccia and colleagues [58] found that increased funding in Massachusetts failed to increase the number of people who received addiction treatment services, even though providers received more dollars per client. The disconnected cycle of treatments may not change until addiction treatment services are organized in to a continuum of care or weaved in to a healthcare system. Moreover, the redundancies observed in our cohort, such as requiring clients to complete an IRS and assessment with each return visit, will continue to disrupt the flow of treatment in the future if left unchanged. UAT and other treatment providers in the State will likely follow these same treatment protocols/funding guidelines with the expanded population of adults who will have Medicaid under the ACA. Organizational and policy changes are needed, in addition to increased funding, to make a significant impact on attrition rates [5].

## Authors’ information

DL is the director of research at the UAT and has been in this role for 11 years. DL is a community psychologist who focuses on translational research in the behavioral health field. DL has overseen multiple federal grant projects at UAT and has extensive experience in archival data collection procedures at the agency.

HD was a research associate at the UAT with over nine years of experience collecting data from the agency’s electronic health records and other sources of data. HD resigned from the agency on August 21, 2014. She has worked in the behavioral health field for over 10 years. HD collected all data elements noted in the manuscript, including billing data, service data from two agency-based database systems, assessment forms from the 1003 clients who completed an assessment, and interviews with agency staff involved in the enrollment process.

## Abbreviations

SUD: Substance use disorder
SAMHSA: Substance Abuse and Mental Health Services Administration
ACA: Accountable Care Act
UAT: Urban Addiction Treatment
IOP: Intensive outpatient (substance use treatment)
IRS: Initial request for services
OP: Outpatient (substance use treatment)
DASA: Division of Alcoholism and Substance Abuse
IL- DHS: Illinois Department of Human Services
DMH: Department of Mental Health.

## References

[1] Fehr BJ, Weinstein SP, Sterling RC, Gottheil E (1992). “As soon as possible” an initial treatment engagement strategy. Subst Abuse.

[2] Siqueland L, Crits-Christoph P, Gallop B, Gastfriend D, Lis J, Frank A, Griffen M, Blaine J, Luborsky L (2002). Who starts treatment: engagement in the NIDA collaborative cocaine treatment study. Amer J Addict.

[3] Carroll K, Carroll KM (1997). Compliance and alcohol treatment: an overview. Improving Compliance in Alcohol Treatment: Project Match Monograph.

[4] Curran GM, Stecker T, Han X, Booth BM (2007). Individual and program predictors of attrition from VA substance use treatment. J Behav Health Serv Res.

[5] Hoffman KA, Ford JH, Tillotson KJ, Choi D, McCarty D (2011). Days to treatment and early retention among patients in treatment for alcohol and drug disorders. Addict Behav.

[6] Substance Abuse and Mental Health Services Administration (SAMHSA): **Results from the 2012 National Survey on Drug Use and Health: Mental health findings**. http://www.samhsa.gov/data/NSDUH/2012SummNatFindDetTables/NationalFindings/NSDUHresults2012.pdf

[7] Edlund MJ, Booth BM, Feldman ZL (2009). Perceived need for treatment for alcohol use disorders: results from two national surveys. Psych Serv.

[8] Grella CE, Karno MP, Warda US, Moore AA, Niv N (2009). Perceptions of need and help received for substance dependence in a national probability survey. Psych Serv.

[9] Tucker JA, Vuchinich RE, Rippens PD (2004). A factor analysis study of influences on patterns of help-seeking among treated and untreated alcohol dependent persons. J Subst Abuse Treat.

[10] Greenfield SF, Brooks AJ, Gordon SM, Green CA, Kropp F, McHugh K, Lincoln M, Hien D, Miele GM (2007). Substance abuse treatment entry, retention, and outcome in women: a review of the literature. Drug Alcohol Depen.

[11] Substance Abuse and Mental Health Services Administration (SAMHSA) Center for Behavioral Health Statistics and Quality: **The TEDS report: age of substance use initiation among treatment admissions aged 18 to 30**. http://www.samhsa.gov/data/2K14/TEDS142/sr142-initiation-age-2014.pdf 27631064

[12] Substance Abuse and Mental Health Services Administration (SAMHSA): **Treatment Episode Data Set (TEDS): 2001–2011. national admissions to substance abuse treatment**. http://www.samhsa.gov/data/2k13/TEDS2011/TEDS2011N.pdf

[13] Garnick DW, Horgan CM, Acedvedo A, Lee MT, Panas L, Ritter GA, Dunigan R, Bidorini A, Campbell K, Haberlin K, Huber A, Lambert-Wacey D, Leeper T, Reynolds M, Wright D (2014). Criminal justice outcomes after engagement in outpatient substance abuse treatment. J Subst Abuse Treat.

[14] Booth BM, Curran GM, Han X, Edlund MJ (2013). Criminal justice and alcohol treatment: results from a national sample. J Subst Abuse Treat.

[15] Dennis ML, Scott CK, Funk R, Foss MA (2005). The duration and correlates of addiction and treatment careers. J Subst Abuse Treat.

[16] Substance Abuse and Mental Health Services Administration (SAMHSA): **Results from the 2011 National Survey on Drug Use and Health: summary of national findings**. http://www.samhsa.gov/data/nsduh/2k11results/nsduhresults2011.pdf

[17] Longhi D, Oatis S, Mudar K, Spaeth D, Van Dyck M, Shaklee M, Brown M, Hall-Milligan J: **The ADASTA program: client services and treatment outcomes**. http://www.dshs.wa.gov/pdf/ms/rda/research/4/17.pdf

[18] Festinger DS, Lamb RJ, Kountz MR, Kirby KC, Marlowe DB (1995). Pretreatment dropout as a function of treatment delay and client variables. Addict Behav.

[19] Chawdhary A, Sayre SL, Green C, Schmitz JM, Grabowski J, Mooney ME (2007). Moderators of delay tolerance in treatment seeking cocaine users. Addict Behav.

[20] Kleinman PH, Kang S, Lipton DS, Woody GE, Kemp J, Millman RB (1992). Retention of cocaine abusers in outpatient psychotherapy. Am J Drug Alcohol Abuse.

[21] Gottheil E, Sterling RC, Weinstein SP, Kurtz JW (1994). Therapist/patient matching and early treatment dropout. J Addict Dis.

[22] Eskhoff D, Radcliff A, Gregory M (1996). The Southern California Kaiser-Permanente chemical dependence recovery program evaluation: results of treatment outcome study in an HMO setting. J Addict Dis.

[23] Rohrer JE, Vaughn MS, Cadoret RJ, Carswell C, Patterson A, Zwick J (1996). Effect of centralized intake on outcomes of substance abuse treatment. Psychiatr Serv.

[24] Vendetti J, McRee B, Miller M, Christiansen K, Herrell J, The Marijuan Treatment Project Research Group (2002). Correlates of pre-treatment drop-out among persons with marijuana dependence. Addiction.

[25] Hser Y, Maglione M, Polinsky ML, Anglin MD (1998). Predicting drug treatment entry among treatment-seeking individuals. J Subst Abuse Treat.

[26] Pena JM, Franklin RR, Rice JC, Foulks EF, Bland IJ, Shervington D, James A (1999). A two-rate hypothesis for patterns of retention in psychosocial treatments of cocaine dependence: findings from a study of African- American men and a review of the published data. Am J Addict.

[27] Weisner C, Mertens J, Tam T, Moore C (2001). Factors affecting the initiation of substance abuse treatment in managed care. Addiction.

[28] Arfken CL, Klein C, diMenza S, Schuster CR (2001). Gender differences in problem severity at assessment and treatment retention. J Subst Abuse Treat.

[29] Donovan DD, Rosengren DB, Downey L, Cox GB, Sloan KL (2001). Attrition prevention with individuals awaiting publicly funded drug treatment. Addiction.

[30] Claus RE, Kindleberger LR (2002). Engaging substance abusers after centralized assessment: predictors of treatment entry and dropout. J Psychoactive Drugs.

[31] Parker JD, Turk CL, Busby LD (2002). A brief telephone intervention targeting treatment engagement from a substance abuse program wait list. J Behav Health Serv Res.

[32] Angarita GA, Reif S, Pirad S, Lee S, Sharaon E, Gastfiend DR (2007). No-show for treatment in substance abuse patients with comorbid symptomatology: validity results from a controlled trial of ASAM patient placement criteria. J Addic Med.

[33] Pinto RM, Campbell AN, Hien D, Yu G, Gorrochurn P (2011). Retention in the National Institute on Drug Abuse Clinical Trials Network Women and Trauma Study: implication for post-trial implementation. Am J Orthopsychiatry.

[34] Resko SM, Mendoza NS (2014). Early attrition from treatment among women with cooccurring substance use disorders and PTSD. J Soc Work Pract Addict.

[35] Molfenter T (2013). Reducing appointment no-shows : going from theory to practice. Subst Use Misuse.

[36] Brown VB, Melchoir LA (2008). Women with co-occurring disorders (COD): treatment settings and service needs. J Psychoactive Drugs.

[37] Ghee AC, Johnson CS, Burlew AK, Bolling LC (2009). Enhancing retention through a condensed trauma-intervention for women with chemical dependence. N Am J Psychol.

[38] Adams SM, Peden AR, Hall LA, Rayens MK, Staten RR, Leukefeld CG (2011). Predictors of retention of women offenders in a community-based residential substance abuse treatment program. J Addict Nurs.

[39] Choi S, Adams SM, MacMaster SA, Seiters J (2013). Predictors of residential treatment retention among individuals with co-occurring substance abuse and mental health disorders. J Psychoactive Drugs.

[40] Substance Abuse and Mental Health Services Administration (SAMHSA), Center for Behavioral Health Statistics and Quality (2013): **Treatment episode data sets (TEDS): 2010 discharges from substance abuse treatment services**. http://www.samhsa.gov/data/DASIS/TEDS2010D_Web.pdf

[41] Carr CJA, Xu J, Redko C, Lane DT, Rapp RC, Goris J, Carlson RG (2008). Individual and system influences on waiting time for substance abuse treatment. J Subst Abuse Treat.

[42] Stark MJ, Campbell BK, Brikerhoff CV (1990). “Hello, may we help you?” a study of attrition prevention at the time of the first phone contact with substance abusing clients. Am J Drug Alcohol Abuse.

[43] Festinger DS, Lamb RJ, Kirby KC, Marlowe DB (1996). The accelerated intake: a method for increasing initial attendance to outpatient treatment for cocaine addiction. J Appl Behav Anal.

[44] Festinger DS, Lamb RJ, Marlowe DB, Kirby KC (2002). From telephone to office: intake attendance as a function of appointment delay. Addict Behav.

[45] Stasiewicz PR, Stalker R (1999). A comparison of three “interventions” on pretreatment dropout rates in an outpatient substance abuse clinic. Addict Behav.

[46] Maddox JF, Desmond DP (1995). Rapid admission and retention on methadone. Am J Drug Alcohol Abuse.

[47] Downey L, Rosengren DB, Donovan DM (2003). Gender, waitlists and outcomes for public sector drug treatment. J Subst Abuse Treat.

[48] McCarty D, Gustafson DH, Wisdom JP, Ford J, Choi D, Molfenter T, Capoccia V, Cotter F (2007). The Network for the Improvement of Addiction Treatment (NIATx): enhancing access and retention. Drug Alcohol Depend.

[49] Brucker DL, Stewart M (2011). Performance based contracting within a state substance abuse treatment system: a preliminary exploration of differences in client access and client outcomes. J Behav Health Serv Res.

[50] Quanbeck A, Wheelock A, Ford JH, Pulvermacher A, Capoccia V, Gustafson D (2013). Examining access to addiction treatment: scheduling process and barriers. J Subst Abuse Treat.

[51] Guerro E (2013). Enhancing access and retention in substance abuse treatment: the role of Medicaid payment acceptance and cultural competence. Drug Alcohol Depend.

[52] Daly DC, Marlat GA (2006). Overcoming your Alcohol or Drug Problem: Effective Recovery Strategies Workbook (Treatments That Work).

[53] Substance Abuse and Mental Health Services Administration (SAMHSA), Center for Behavioral Health Statistics and Quality (2012): **Treatment episode data sets (TEDS): 2000–2010 state admissions to substance abuse treatment services**. http://www.samhsa.gov/data/2k12/TEDS2010N/TEDS2010NWeb.pdf

[54] Illinois Department of Human Services: **Division of alcoholism and substance abuse automated reporting and tracking system (DARTS)**. http://www.dhs.state.il.us/onenetlibrary/27896/documents/manuals/fy12/darts_manual_for_fy2012.pdf

[55] Buck JA (2011). The looming expansion and transformation of public substance abuse treatment under the Affordable Care Act. Health Aff.

[56] Mark TL, Levit KR, Vandivart-Warren R, Buck JA, Coffey RM (2011). Changes in US spending on mental health and substance abuse treatment, 1986–2005, and implications for policy. Health Aff.

[57] Tai B, Volkow ND (2013). Treatment for substance use disorders: opportunities and challenges under the affordable care act. J Soc Work Public Health.

[58] Cappocia VA, Grazier KL, Toal C, Ford JH, Gustafson DH (2012). Massachusett’s experience suggest coverage alone is insufficient to increase addiction disorders treatment. Health Aff.

